# The role of HbA1c in the bidirectional relationship between periodontitis and diabetes and related interventions: a narrative review

**DOI:** 10.3389/fnut.2025.1606223

**Published:** 2025-06-17

**Authors:** Yudie Du, He Xiao, Ruihan Luo, Guangyi Li, Yixing Ren

**Affiliations:** ^1^Department of Gastrointestinal Surgery, Affiliated Hospital of North Sichuan Medical College, Nanchong, China; ^2^Institute of Hepatobiliary Pancreatic Intestinal Diseases, North Sichuan Medical College, Nanchong, China; ^3^Department of General Surgery, Chengdu XinHua Hospital Affiliated to North Sichuan Medical College, Chengdu, China

**Keywords:** diabetes mellitus, periodontitis, glycated hemoglobin, non-surgical periodontal therapy, photodynamic therapy

## Abstract

**Objective:**

The intricate relationship between periodontitis and diabetes mellitus (DM) has emerged as a focal point in contemporary medical research. This study aimed to explore the role of glycated hemoglobin (HbA1c) in this relationship, examine the mechanistic theories that may underlie this connection, and summarize effective interventions.

**Methods:**

Articles were retrieved from PubMed/Medline and Web of Science. All studies focusing on this bidirectional relationship were included and evaluated.

**Results:**

Several mechanism theories have been proposed, including alterations in oral flora, suppression of anti-inflammatory and antidiabetic control mechanisms in periodontitis, gene silencing due to reduced DNA demethylation, and significantly lower quantitative levels of platelet-rich fibrin (PRF). Non-surgical periodontal therapy (NSPT) is an effective intervention, and based on this, systemic antibiotics and propolis can also be used. Antimicrobial photodynamic therapy (aPDT) is also an adjunct to periodontal therapy, with indocyanine green currently receiving increased attention.

**Discussion:**

HbA1c links periodontitis and DM bidirectionally, with elevated levels contributing to the worsening of periodontal disease and tooth loss. Studies have confirmed this association in both diabetic and non-diabetic individuals, although some findings remain conflicting. While HbA1c may predict diabetes risk and periodontal severity, further research is needed to clarify this relationship.

**Conclusion:**

There is a bidirectional relationship between periodontitis and DM, with HbA1c playing an important role in this relationship. However, this study has certain limitations regarding selection bias and methodology, necessitating further mechanistic exploration and clinical validation in subsequent research.

## 1 Introduction

The exploration of all aspects of type 2 diabetes (T2D), glycated hemoglobin (HbA1c), periodontitis, and chronic periodontitis treatments has remained an enduring and consistent focus over time. These topics have remained highly prominent in the literature, with their significance being repeatedly highlighted across different periods (1).

Diabetes mellitus (DM) is a metabolic condition marked by abnormally high blood glucose levels, which arise due to either inadequate insulin production or the body’s reduced ability to effectively utilize insulin (2). It is commonly linked to various systemic complications, such as vision impairment, kidney dysfunction, nerve damage, and an increased risk of cardiovascular disorders (3). DM significantly exacerbates the development of atherosclerotic processes and is strongly correlated with elevated risks of coronary artery disease, cerebrovascular events, and overall mortality rates (4). It has emerged as a global pandemic, ranking among the most prevalent chronic health conditions worldwide. Recent epidemiological data reveal that approximately 10.5% of the global adult population (equating to 536.6 million individuals) was affected by diabetes in 2021. Current projections suggest a concerning upward trend, with estimates indicating this figure will escalate to 12.2% of the world’s adult population (approximately 783.2 million individuals) by the year 2045 (5). T2DM represents the predominant form of diabetes, constituting an estimated 90–95% of total diabetes cases worldwide (6).

HbA1c, representing the percentage of glucose-bound hemoglobin in circulation, has been established as a reliable predictor of DM-associated complications. This clinically validated biomarker is integral to both diagnostic protocols and therapeutic monitoring, serving as a quantitative measure of glycemic control and treatment efficacy in DM management (7). A comprehensive longitudinal investigation conducted within the American Indian population established HbA1c as a robust predictive marker for diabetes development. The study revealed a substantially elevated diabetes incidence rate among pediatric subjects with prediabetic conditions when compared to their normoglycemic counterparts (8). Furthermore, the Japanese clinical guidelines for diabetes set the cut-off value for HbA1c at less than 6.3% (9).

**Figure 1:**
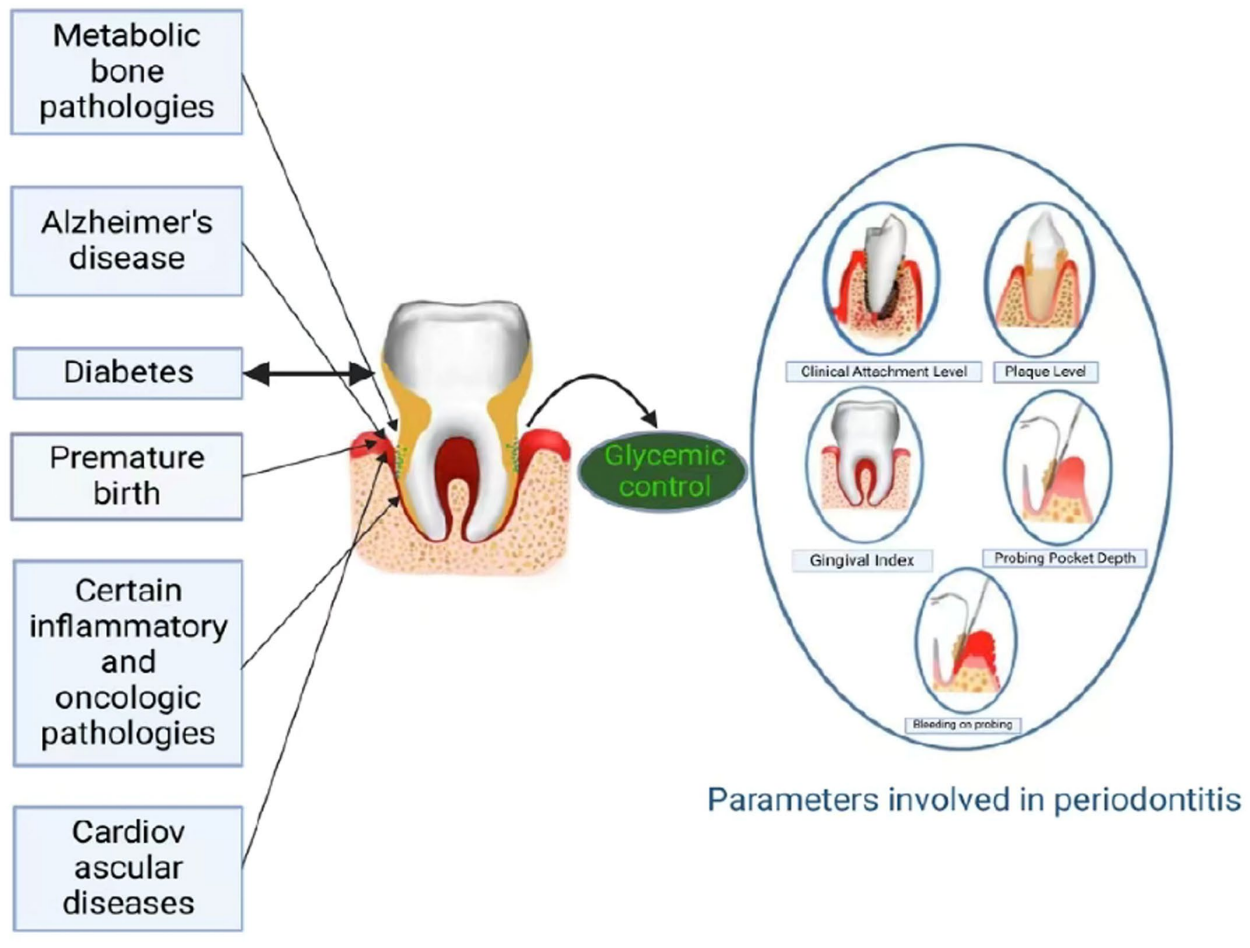
Inducement and parameters of periodontal disease.

Periodontitis represents a globally prevalent chronic disorder, characterized by both infectious and immune-mediated inflammatory components, making it one of the most widespread oral health conditions worldwide (10). Periodontitis is marked by the formation of periodontal pockets, detachment of gingival tissue, and alveolar bone loss beneath the soft tissue (11). It arises from a complex interaction among specific Gram-negative microorganisms, their byproducts, and the host’s tissue response (12). Periodontitis can be diagnosed using a variety of indices, including probing pocket depth (PPD), bleeding on probing (BOP), tooth mobility, tooth loss, clinical attachment loss (CAL), and radiographic analysis of alveolar bone morphology (Figure 1). The diagnosis is made by integrating these findings (13, 14). Extensive epidemiological research has established significant associations between advanced periodontitis and a spectrum of 43 systemic conditions, encompassing cardiovascular pathologies, pregnancy complications (including preterm delivery and fetal growth restriction), respiratory infections, autoimmune disorders such as rheumatoid arthritis, and metabolic liver diseases including non-alcoholic steatohepatitis (15–17), which is also shown in Figure 1.

Although the relationship between periodontitis and DM is likely bidirectional (18, 19), there are still studies that do not confirm this finding (20). Therefore, this study aims to summarize the current complex relationship between periodontitis and DM, presenting its impact through mechanisms such as inflammation, genetics, and microbial imbalance. Furthermore, this study also examines the important role of HbA1c in this relationship and suggests currently effective interventions, with the aim of providing new perspectives on slowing the progression of DM and periodontitis.

## 2 Methods

A comprehensive literature search of PubMed/Medline and Web of Science was conducted to determine the bidirectional relationship between periodontitis and DM. The following search terms were used: “diabetes mellitus,” “periodontitis,” “glycated hemoglobin,” “non-surgical periodontal therapy,” “photodynamic therapy,” and “mechanisms.” The search terms were applied to the database without restrictions on publication date or study type. Two researchers independently screened the titles and abstracts based on the inclusion criteria. After discussion to reach a consensus on eligible articles, the selected papers were thoroughly reviewed and analyzed in alignment with the study objectives.

The inclusion criteria for the literature search comprised: meta-analyses, clinical trials, mechanistic studies, and English-language reviews. The selection criteria assessed: clinical trials involving animal or human subjects, *in vitro* cellular investigations, and sophisticated physicochemical analyses.

## 3 Results

### 3.1 Mechanisms that may be related to two-way relationships

#### 3.1.1 Chronic inflammation and oral microbiota imbalance

Emerging research indicates that the association between diabetes and oral health is underpinned by intricate biological pathways, including persistent inflammatory responses, and alterations in the composition of oral microbial communities (21–23). The persistent pro-inflammatory state characteristic of diabetes exacerbates oral inflammatory processes, while dysbiosis of the oral microbiome creates an environment conducive to the proliferation of pathogenic microorganisms. Furthermore, diabetes-induced modifications in salivary gland function and compromised tissue regeneration capacity exacerbate oral health deterioration (24, 25). The underlying mechanism involves the deterioration of saliva’s natural protective properties in diabetic patients. Specifically, the impairment of saliva’s cleansing efficiency and acid-neutralizing capacity leads to significant alterations in oral ecology. The consequent reduction in salivary pH fosters an environment conducive to the growth of acid-resistant microorganisms, which, in turn, promotes the dominance of acid-generating bacterial populations. This microbial transition results in an unfavorable ecological niche that suppresses beneficial oral microbiota (26). Notably, the reciprocal interplay between diabetes and oral health status underscores the necessity for integrated oral-systemic healthcare approaches, emphasizing both optimized metabolic regulation and specialized periodontal management to improve clinical outcomes in diabetic patients. This further supports the concept that diabetes and periodontitis are closely interconnected.

#### 3.1.2 Altered expression levels of periodontal pathogens

In the clinical trial, researchers observed significant changes in the expression levels of *Fusobacterium nucleatum* (Fn) and *Tannerella forsythia* (Tf) in patients with diabetes compared to healthy controls. Additionally, Fn and Tf levels were found to positively correlate with fasting blood glucose and glycated hemoglobin (HbA1c) levels. Notably, Fn and Tf are key microbiota associated with periodontitis (27). Moreover, they investigated and clarified the molecular mechanism by which *Fusobacterium nucleatum* culture filtrate (FNCF) induces cytokine release through the Toll-like receptor 2 (TLR2) signaling pathway in human gingival epithelial Smulow–Glickman (S–G) cells. The use of an extracellular signal-regulated kinase (ERK) inhibitor (U0126) demonstrated that FNCF regulates the insulin receptor substrate 1 and protein kinase B (IRS1/AKT) signaling pathway. This regulation affects key proteins involved in hepatic glycogen synthesis, including glycogen synthase kinase-3 beta (GSK3β) and glycogen synthase (GS), ultimately contributing to insulin resistance (28). These findings suggest that the expression products of Fn affect hepatic glycogen synthesis, leading to elevated blood glucose levels.

#### 3.1.3 Altered levels of DNA methylation in genes associated with inflammation

Epigenetics—which refers to changes in gene expression without altering the DNA sequence (29)—also contributes to the pathogenesis of periodontitis (30). One of the primary epigenetic mechanisms is DNA methylation, which involves the addition of methyl groups to the 5′ position of the cytosine base (5mC), predominantly occurring at CpG sites within gene promoters. DNA methylation typically leads to gene silencing, while DNA demethylation enables gene expression. DNA methyltransferases (DNMTs) are the enzymes responsible for catalyzing the methylation of DNA (29). Interestingly, studies have revealed that 5mC can be further converted into 5-hydroxymethylcytosine (5hmC) through a reaction regulated by 10-11 translocation (TET) proteins (31). Although the conversion to 5hmC is catalyzed by TET enzymes, this reaction requires the cofactor *α*-ketoglutarate (α-KG), which is produced by isocitrate dehydrogenases (IDHs) (32). The formation of 5hmC leads to DNA demethylation. Research in the field of periodontology has identified altered DNA methylation levels in genes related to inflammation (33, 34). The TET2 AA and AC genotypes have been reported to be associated with periodontitis. Additionally, the AA genotype has been linked to elevated levels of HbA1c (35). TET2 is a crucial regulator of 5mC demethylation and is capable of oxidizing 5mC in RNA within innate immune cells (36). In one study, it was observed that *α*-KG and HbA1c were positively correlated (37). α-KG plays a crucial role in the TET2-mediated process of converting 5hmC back to cytosine, thereby facilitating DNA demethylation (31). Individuals with diabetes and an HbA1c level of approximately 10% exhibited reduced levels of 5hmC compared to healthy controls, a finding attributed to the destabilization of TET2 caused by hyperglycemia (38). It can be deduced that hyperglycemia leads to TET2 instability, and 5hmC levels are reduced in diabetics, so DNA demethylation is reduced and genes are silenced. This has been linked to the observation of aberrant promoter methylation profiles of genes involved in inflammatory activation in gingival tissue, peripheral blood, or buccal mucosa of patients with periodontitis. This mechanism may explain the complex relationship between periodontitis and diabetes.

A recent investigation analyzed the allelic distribution of the G > A polymorphism in DNMT1 SNP rs2288349, an intronic variant situated on the antisense DNA strand. The study revealed a statistically significant association between the AG and AA genotypes of this polymorphism and reduced susceptibility to periodontitis development (35). This suggests that the A allele may be “protective” against periodontitis, although the specific mechanism by which the genotype affects DNMT1 function remains unclear. Further studies should investigate their encoding genes and their combined influence on periodontitis to better understand the mechanisms behind its pathogenesis and progression. Both *in vitro* and *in vivo* experimental models have demonstrated that *Porphyromonas gingivalis* can influence DNMT1 expression and that DNMT1 may exert protective effects against periodontitis (39, 40). Notwithstanding these findings, comprehensive mechanistic investigations are warranted to delineate the precise involvement of DNMT1 in the pathogenesis of periodontitis and to characterize the functional implications of the rs2288349 polymorphism on its regulatory mechanisms.

#### 3.1.4 Suppression of immunoregulatory mechanisms

Panezai et al. (41) reported a significant finding regarding the reduced immunoregulatory mechanisms and elevated glycemia influenced by periodontitis. HbA1c was found to be inversely associated with LAP-TGF-beta, FGF-19, and IL-10, indicating both diabetogenic and hyporesponsive immune mechanisms. FGF-19 operates through a delayed feedback mechanism to modulate pancreatic endocrine function, specifically by stimulating the coordinated secretion of both insulin and glucagon, thereby contributing to the maintenance of systemic glucose equilibrium (42). The latency-associated peptide (LAP) binds to TGF-*β* as a proprotein, rendering it inactive. The resulting LAP/TGF-β complex is highly expressed in regulatory T cells (43). As a potent immunoregulatory cytokine, IL-10 exerts its biological functions through dual mechanisms: (1) downregulation of proinflammatory cytokine signaling cascades and (2) modulation of T cell-mediated immunological pathways, thereby maintaining immune homeostasis (44). Previous research by Panezai et al. has established a significant correlation between impaired T-cell functionality and reduced concentrations of the immunoregulatory cytokine IL-10 with the progression and severity of periodontal disease (45). At the same time, *in vitro* studies have shown that under hyperglycemic conditions, the anti-inflammatory efficacy of IL-10 is diminished, consequently affecting IL-10-mediated signaling pathways (46). This report therefore suggests that the dysregulation of glucose homeostasis and suppression of the immune response may occur under the influence of periodontitis.

#### 3.1.5 Significant reduction in PRF levels

An *ex vivo* study revealed that among patients with periodontitis, there was a moderate, negative, and significant correlation between HbA1c levels and the quantity of Platelet-rich Fibrin (PRF) obtained. Notably, a significant inverse correlation was observed between HbA1c levels and PRF quantity, with PRF demonstrating a marked reduction as HbA1c concentrations increased (47). It was further observed that the amount of PRF obtained was significantly higher in non-diabetic patients compared to diabetic patients. PRF, which stands for platelet-rich fibrin, is an autologous second-generation platelet concentrate. It is currently being used as an engineered scaffold to enhance the body’s natural wound-healing mechanisms (48). In a recent study, Gupta et al. (49) suggested that the structure and cytokine profile of PRF may be altered in patients with periodontitis and diabetes. Whether these alterations are causally related to the development of periodontitis and diabetes remains to be explored through a large number of experiments.

### 3.2 Interventions to impede two-way relationships

**Figure 2:**
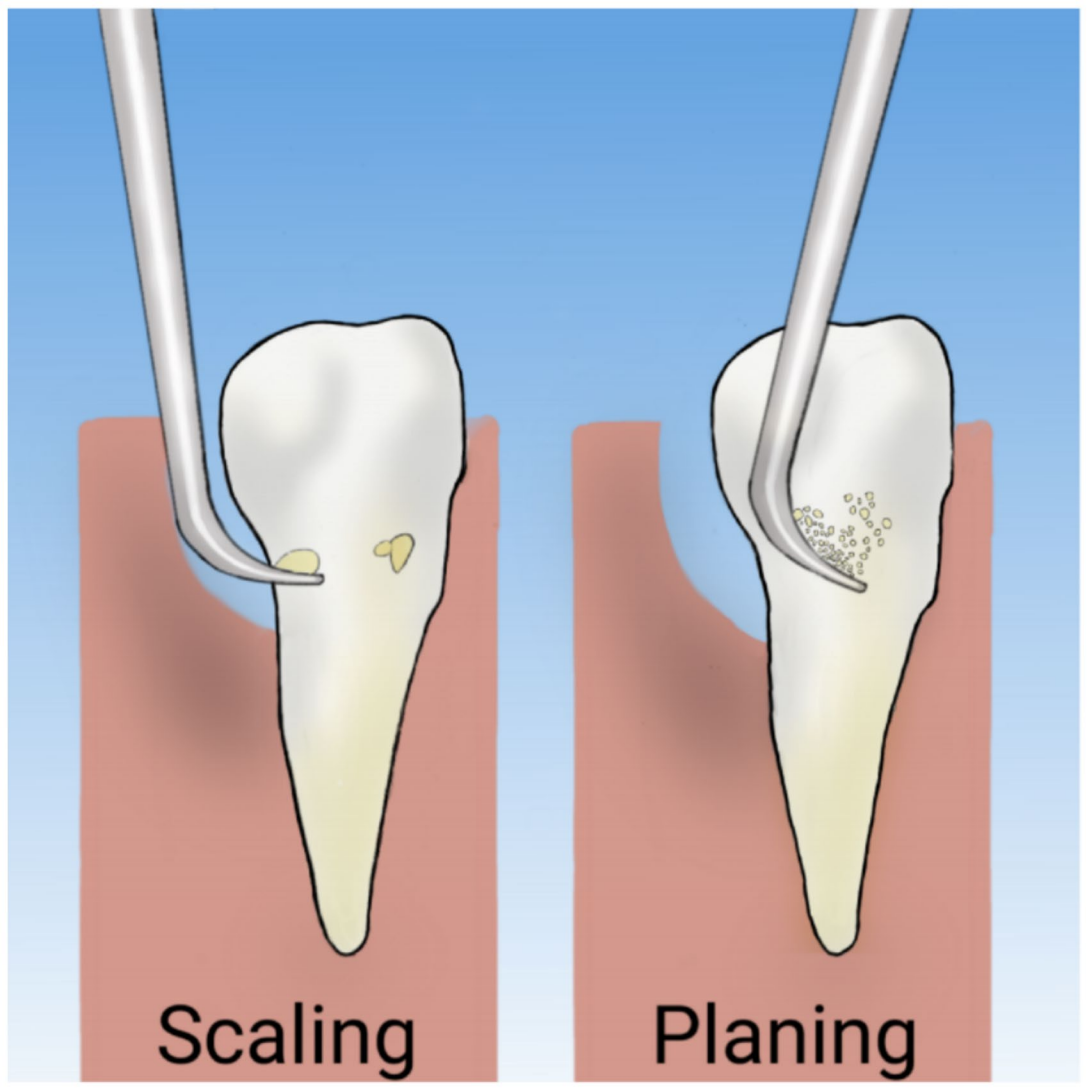
Scaling and root planing.

Periodontal diseases are typically treated either through surgical intervention or non-surgical periodontal therapy (NSPT). A 2022 update of a Cochrane review on periodontal management for diabetic patients reported a moderate level of evidence for improved glycemic control up to 1 year following NSPT (50). Individuals with diabetes and periodontitis experienced a significant improvement in glycemic control compared to those who remained untreated or received standard care. Results from another review, which included data from 30 studies, showed that in patients with diabetes, there was a 0.43% reduction in HbA1c levels 3 to 4 months after periodontal treatment, compared to those receiving routine care or no intervention. In 12 trials with a 6-month follow-up, this difference was 0.30% (51), whereas one study revealed a 0.5% reduction in HbA1c after 12 months (50). Scaling and root planing (SRP) represents the fundamental therapeutic approach in non-surgical periodontal treatment (Figure 2), functioning through mechanical disruption of subgingival biofilm architecture to achieve substantial reduction or complete eradication of pathogenic bacterial colonies (52). One study demonstrated that SRP alone could reduce HbA1c levels by as much as 0.72% at 3 months post-intervention (53), which demonstrates comparable efficacy to a 3-month regimen of 1,000 mg extended-release metformin administered once daily (54). The magnitude of HbA1c reduction demonstrated a temporal decline, with the 6-month outcomes showing less pronounced improvement compared to the 3-month results. This temporal pattern aligns with existing evidence suggesting an inverse correlation between baseline HbA1c levels and therapeutic response, where patients with elevated initial HbA1c values tend to exhibit more substantial reductions following non-surgical periodontal interventions (55). For this reason, patients with poor glycemic control are strongly advised to undergo SRP every 3 months, which may lead to significant improvements in glycemic control.

A meta-analysis evaluated whether systemic antibiotics, used as an adjuvant to SRP, could benefit periodontal patients with T2D. Therapeutic outcomes demonstrated that both SRP monotherapy and SRP combined with adjunctive systemic antibiotics (SRPa) achieved significant reductions in both PPD and HbA1c levels at the 3-month post-treatment evaluation in T2D patients with periodontitis. However, at the 6-month assessment, while SRP maintained its efficacy in reducing both parameters, SRPa showed limited effectiveness, demonstrating PPD reduction but failing to sustain HbA1c improvement (53). To further investigate the impact of SRPa treatments on HbA1c reduction at the 6-month follow-up, we anticipate more randomized controlled trials (RCTs) with extended follow-up periods after SRP or SRPa treatments. Moreover, another study demonstrated that SRP combined with doxycycline is more effective in improving HbA1c levels among patients with T2D and periodontitis compared to SRP alone. Most of the improvement is observed within the first 3 months of follow-up (56).

A network meta-analysis investigated the effects of propolis supplementation alongside NSPT and found that it likely improves HbA1c levels in patients with T2D and periodontitis, showing a large effect with moderate certainty. Additionally, supplementation with alpha lipoic acid and melatonin may also help reduce HbA1c levels in T2D patients with periodontitis, though these results show large effects with low certainty (57). Due to its antimicrobial properties, propolis is often referred to as a natural antibiotic (58). Polyphenols, which are found in propolis, have been proposed as effective compounds that may help prevent and manage T2D. They are believed to enhance glucose metabolism, reduce insulin resistance and HbA1c levels, and improve vascular function (59). Furthermore, propolis can be beneficial in the treatment of periodontitis, enhancing the outcomes of NSPT due to its anti-inflammatory, antibacterial, and antioxidant properties (58, 60). The use of this substance may reduce the prevalence of periodontal pathogens, such as *Porphyromonas gingivalis*, *Prevotella intermedia*, and *Fusobacterium nucleatum*, and potentially improve periodontal parameters when used as an adjunct to NSPT (58).

**Figure 3:**
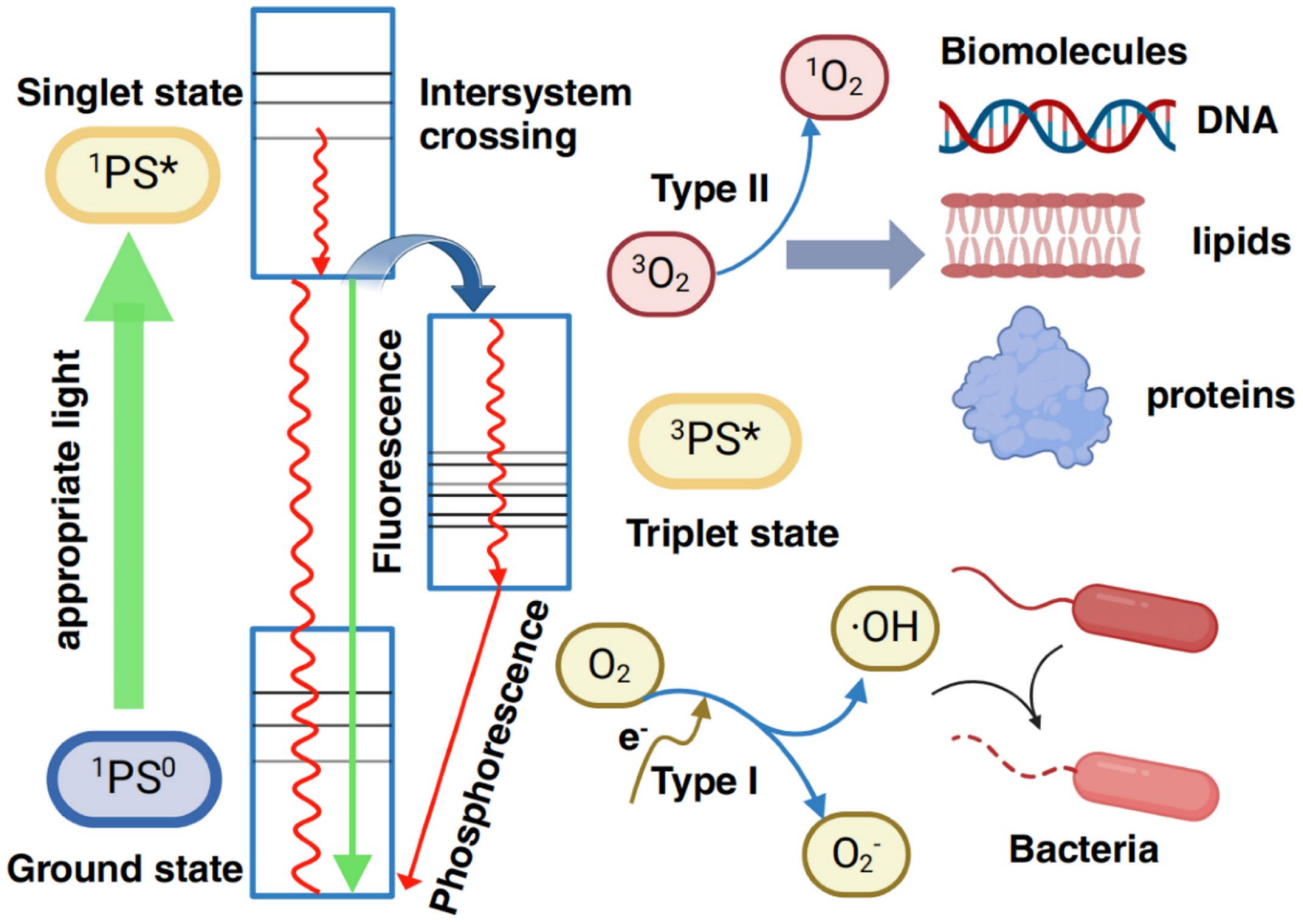
Mechanism of aPDT. PS, photosensitizer.

Antimicrobial photodynamic therapy (aPDT) is an adjunctive, minimally invasive therapeutic approach (61), and has emerged as an additional method for periodontal treatment, serving as an alternative to antibiotic therapy, which can cause a number of side effects, including toxicity and the emergence of resistant microorganisms. aPDT utilizes specific wavelengths of light to activate photosensitizers, which subsequently produce reactive oxygen species (ROS) such as singlet oxygen (^1^O₂) and hydroxyl radicals (·OH). These ROS induce oxidative damage to essential biomolecules, including phospholipids, proteins, and DNA, within microorganisms, ultimately leading to cell death. Photosensitizers transition between excited singlet and triplet states, enabling the production of ROS through two mechanisms: Type I reactions (involving electron transfer to generate free radicals) and Type II reactions (involving energy transfer to produce singlet oxygen). To overcome the limitations of traditional photosensitizers, such as poor water solubility, photobleaching, and low selectivity, novel photosensitizers have been developed. These include aromatic small molecules, conjugated polymers, and nanomaterials. Additionally, multifunctional systems such as chemiluminescent excitation, smart responsive delivery, and oxygen self-enriching systems have been created. These advancements address challenges such as the limited tissue penetration depth of light and the hypoxic conditions often found at infection sites, significantly improving the antimicrobial effectiveness and clinical potential of aPDT (62) (Figure 3).

The majority of clinical investigations exploring aPDT applications in periodontitis management have predominantly utilized photosensitizers from three major classes: phenothiazine derivatives (including methylene blue and toluidine blue), xanthene compounds, and riboflavin-based agents. In recent years, the scientific focus has shifted toward indocyanine green, with emerging evidence highlighting its superior photosensitizing properties and therapeutic potential in periodontal applications (63). Indocyanine green has been extensively characterized as a biocompatible agent with a favorable safety profile, demonstrating none of the adverse effects typically associated with conventional antibiotic therapies (63), and its mechanism of action differs from that of other photosensitizers—it exhibits a 20% photodynamic effect, with its primary action being through a photothermal effect. This effect induces cell damage by raising the intracellular temperature (64). Photothermal therapy involves the absorption of energy from laser radiation by indocyanine green, leading to a significant increase in local temperature (65). *In vitro* studies have demonstrated that aPDT using indocyanine green can effectively reduce the bacterial load in periodontal pockets (66, 67). Boehm and Ciancio (67) demonstrated that aPDT using indocyanine green significantly reduced the viability of *A. actinomycetemcomitans* and *P. gingivalis*. Srikant et al. (68) observed a significant reduction in the proportion of viable bacteria at the end of 1 week in sites treated with indocyanine green (5 mg/mL) aPDT, compared to sites that underwent SRP alone or SRP combined with low-level laser therapy. Scientific investigations have demonstrated that indocyanine green possesses a dual-action mechanism, complementing its well-documented photothermal properties with significant photodynamic activity mediated through the generation of reactive oxygen species (ROS) (69). HbA1c levels significantly decreased in both groups that underwent scaling and root planing alone and those that received SRP combined with aPDT. However, aPDT did not produce more significant effects on HbA1c compared to SRP alone (70). Therefore, this type of adjunctive therapy has the potential to provide additional clinical benefits to SRP in terms of improving the periodontal condition of patients with type II diabetes. However, it does not offer significant benefits to SRP in terms of glycemic control.

## 4 Discussion

HbA1c can have an important role in the bidirectional relationship between periodontitis and diabetes mellitus. Costa et al. examined how HbA1c levels affect the progression of periodontal disease. Their study demonstrated that the progression of periodontal disease correlates with elevated HbA1c levels in hyperglycemic patients. Additionally, the severity of periodontitis was found to be closely related to increased HbA1c levels, both in individuals with and without type 2 diabetes (71). Furthermore, Costa et al. (72) demonstrated a significant positive association between elevated HbA1c levels and both the clinical progression of periodontitis and the incidence of tooth loss (odds ratio [OR] = 2.9). Similarly, Demmer et al. (73) found that patients with severe hyperglycemia had up to a 3-fold higher risk of tooth loss, and the risk ratio (RR) (95% CI) is 1.36 (1.11–1.67). Interestingly, the association between periodontitis and HbA1c levels in non-diabetic populations remains relatively underexplored, with limited empirical evidence available in the current scientific literature. In a preliminary investigation by Wolff et al. (74), multivariate analysis revealed significantly elevated mean HbA1c levels among periodontitis patients relative to periodontally healthy controls, even after controlling for potential confounding factors (between-group difference, 0.21%; *p* = 0.046). While the observed difference in HbA1c levels between the study groups was clinically modest, it reached statistical significance. This finding is substantiated by a comprehensive population-based cohort study, which demonstrated a significant positive association between baseline chronic periodontal infection status and longitudinal HbA1c level fluctuations (75). The cumulative evidence from current research suggests that HbA1c measurement may represent a valuable diagnostic tool with dual clinical applications: predicting diabetes risk in periodontitis-affected individuals and assessing periodontal disease progression in diabetic patients. Supporting this observation, a recent cross-sectional investigation demonstrated a dose–response relationship, with significantly greater HbA1c level alterations observed in patients presenting with advanced stage III/IV periodontitis compared to those with less severe forms of the disease (95% CI: 0.14–1.56, *p* = 0.02) (76). Further research has established that suboptimal glycemic control, defined as sustained HbA1c levels exceeding 7%, significantly impacts oral health status. Specifically, this metabolic dysregulation has been shown to elevate the relative risk of developing periodontal disease by 2.8-fold and predispose individuals to a 4.2-fold increase in alveolar bone resorption (77). However, in contrast to the prevailing scientific consensus, a longitudinal investigation conducted by Kebede et al. (20) yielded null findings, demonstrating no statistically significant association between periodontitis status and diabetes incidence throughout their 11-year prospective cohort study. The analysis failed to demonstrate any significant association between HbA1c level fluctuations and initial periodontal conditions. There are two potential explanations for these unexpected findings: (1) selection bias due to differential survival rates, wherein periodontitis patients with heightened diabetes susceptibility had lower study completion rates; and (2) limited inflammatory progression, as baseline central obesity was more prevalent among participants who subsequently developed diabetes or prediabetic conditions (20).

Currently, numerous experimental studies have demonstrated the bidirectional relationship between periodontitis and diabetes, highlighting the significant role that alterations in HbA1c play in this relationship. However, there are still some studies that fail to report these results. Furthermore, although this study highlights the potential of HbA1c as a dual diagnostic tool, it did not assess its sensitivity or specificity. It also relied primarily on observational study designs, such as cross-sectional and cohort studies, which could not establish causality and could only suggest correlation. Therefore, further meta-analyses and clinical trials are needed to explore whether such a true bidirectional relationship truly exists between periodontitis and diabetes.

## 5 Conclusion

Many studies have confirmed the bidirectional relationship between periodontitis and DM, with HbA1c playing an important role in this relationship. However, some studies have not confirmed this finding, possibly due to limited inflammatory progression or selection bias related to differential survival rates. With such conflicting findings, more meta-analyses and clinical trials are needed in the future to address the complex relationship between the two. The possible alteration of PRF structure and cytokine profile in patients with diabetes mellitus and periodontitis is an interesting observation. More mechanistic studies and clinical trials are needed to determine whether *in vivo* supplementation with normally structured PRF can effectively treat periodontitis. To slow the progression of both periodontitis and diabetes, NSPT is an effective approach. Based on this, systemic antibiotics and propolis have shown additional therapeutic benefits. As a new therapeutic tool, aPDT has demonstrated promising clinical benefits in treating periodontal conditions in diabetic patients; however, more clinical studies are needed to confirm its efficacy in controlling blood glucose. Due to the limitations of this study, such as potential publication bias or heterogeneity within the study population, more relevant studies with effective strategies are needed in the future to slow down the progression of periodontal disease and diabetes.
